# Investigation on the adsorption of phosphorus in all fractions from sediment by modified maifanite

**DOI:** 10.1038/s41598-018-34144-w

**Published:** 2018-10-23

**Authors:** Zisen Liu, Yi Zhang, Fan Han, Pan Yan, Biyun Liu, Qiaohong Zhou, Fenli Min, Feng He, Zhenbin Wu

**Affiliations:** 1 State Key Laboratory of Freshwater Ecology and Biotechnology, Institute of Hydrobiology, Chinese Academy of Sciences, Wuhan, 430072 China; 2 University of Chinese Academy of Sciences, Beijing, 100049 China; 3 School of Resources and Environmental Engineering, Wuhan University of Technology, Wuhan, 430070 China

## Abstract

Sediment phosphorus (P) removal is crucial for the control of eutrophication, and the *in-situ* adsorption is an essential technique. In this study, modified maifanite (MMF) prepared by acidification, alkalization, salinization, calcination and combined modifications, respectively, were first applied to treat sediment P. The morphology and microstructure of MMF samples were characterized by X-ray fluorescence (XRF), Fourier transform infrared (FTIR), X-ray diffraction (XRD), scanning electron microscope (SEM) and Brunauer-Emmett-Teller (BET). Various adsorption parameters were tested, such as dosage of maifanite, time, operation pH and temperature. The adsorption mechanisms were also investigated and discussed. Results showed that CMMF-H2.5-400 (2.5 mol/L H_2_SO_4_ and calcined at 400 °C) exhibited the highest P adsorption capacity. Thus, it was selected as the *in-situ* adsorbent material to control the internal P loading. Under the optimal conditions of dynamic experiments, the adsorption rates of TP, IP, OP, Fe/Al-P and Ca-P by CMMF-H2.5-400 were 37.22%, 44.41%, 25.54%, 26.09% and 60.34%, respectively. The adsorption mechanisms analysis revealed that the adsorption of P onto CMMF-H2.5-400 mainly by ligand exchange. Results of this work indicated that the modification treatment could improve the adsorption capacity of maifanite, and CMMF-H2.5-400 could be further applied to eutrophication treatment.

## Introduction

Eutrophication has become a worldwide water quality issue^1,2^. Phosphorus (P) is an essential element that can trigger severe eutrophication^3,4^. Many recent reports have shown that both nitrogen (N) and P, which are the key limiting nutrients in eutrophication play a dominant role in aquatic ecosystem^5,6^. Accordingly, P limitation occurs in spring, while N becomes the limiting factor in summer and fall^7^. The change in the dual limitation paradigm is because the P concentration in water bodies can be sustained by the release of P from sediments, whereas loss of N through denitrification often occur in warm season^8^. The release of sediment P, also known as internal P loading, has been recognized that the supply of sediment P to the growth of algae has two phases, which is dominated by the cycling of Iron (Fe)-bound P (Fe-P) and algal degradation. The internal loading of P from sediments plays a primary role in seasonal N limitation for harmful algal bloom (HAB), which in turn can drive the release of sediment P^9^. Fe is a redox-sensitive element and its redox cycle play a vital role in controlling the P mobility in sediments^10^. Some recent studies revealed that the formation of anoxic condition at the sediment-water interface during HAB events (HABs), causes the reductive dissolution of Fe (III) oxyhydroxides and the release of Fe-P^11,12^. Furthermore, the algal degradation appears during HABs, resulting in the release of P from the degraded algal cells^13^. In addition, previous studies found that the distribution of soluble reactive P (SRP) and labile P stratified into two layers, called static layer and active layer. In static layer, the concentration of soluble reactive phosphate and labile P remained at a low level, which is the key to sustain the immobilization effectiveness^14^. The active layer not only retains P liberated from the underlying old sediment, but also draws P out of the overlying fresh sediment, thus reducing P release from the sediment^15,16^. More recently, the use of high-resolution dialysis (HR-Peeper) and diffusive gradient in thin films (DGT) techniques for investigating the P mobilization processes in sediments had achieved great progress^9,14,17,18^.

The sediment plays a great role in the overall P dynamics of water bodies-it may either act as a sink to adsorb P or an internal source to release P on the base of concentration gradients^19^. Reducing internal P loading in sediments has been proposed as an essential technique for eutrophication control^20,21^.

The techniques for reducing the release of sediment P are either *ex-situ* or *in-situ* techniques. Sediment dredging, which could reduce P from sediment permanently, has been widely applied to control sediment P in lakes^22,23^. Nevertheless, dredging may destroy the lake ecosystems and enhance the risk in contaminants resuspension, nor are they cost-effective. Therefore, *in-situ* techniques for reducing the sediment internal P loading have received increasing attention in recent years.

Many *in-situ* techniques have been applied to reduce internal P loading in sediment, including *in-situ* capping^24,25^, hypolimnetic aeration^26,27^, phytoremediation^28,29^, and *in-situ* physicochemical adsorption^30,31^, etc. Among these, *in-situ* physicochemical adsorption turns out to be an efficient technique for controlling eutrophication in lakes. The selection of an effective *in-situ* adsorbent materials, which should be environmentally friendly, geographically available, and economically feasible, is critical for the *in-situ* physicochemical adsorption technology. Some natural or modified clay minerals selected as P adsorbent materials have been applied to reduce the sediment internal P loading.

Maifanite is a kind of natural clay minerals with an eco-friendly characteristic, which is abundant in China^32,33^. Maifanite has great surface area and strong adsorption capacity due to the vesicular pores and sponge shaped morphological structure^34,35^. It could adsorb P via chemisorption due to the aluminum (Al) and iron (Fe), etc. in the maifanite^36^. The market price of raw maifanite (RMF) is about 300–400 yuan (¥)/t. Due to its low cost and high capability, maifanite has been applied to soil improvement^35^, which contains amino acid and some trace elements, and could be further combined with ecological remediation. The adsorption capacity of RMF material is usually limited, attributed to the presence of impurity and the permanent negative charges due to isomorphous substitution effect^37^. Thus, there is a need to modify RMF to enhance its confined capacity of adsorption.

Here, RMF granules were modified by acidification, alkalization, salinization, calcination and combined modifications, respectively. Modified maifanite (MMF) granules were prepared and tested as promising *in-situ* physicochemical adsorbent materials to adsorb sediment P in all fractions. This study investigated the adsorptive effects of sediment P in all fractions with MMF for the first time. The specific objectives of the study were to explore the optimal modification methods of maifanite applied as adsorbent material and investigate the adsorption effects and mechanisms of MMF on sediment P in West Lake, Hangzhou, China.

## Results and discussion

### Analysis of sediment characteristics

The quantities of TP, IP, OP, Fe/Al-P and Ca-P in sediment from West Lake were 1652.31 mg·kg^−1^, 1022.29 mg·kg^−1^, 630.02 mg·kg^−1^, 494.45 mg·kg^−1^ and 499.79 mg·kg^−1^, respectively. This illustrated the internal P loading, which was mainly due to the quantities of Fe/Al-P and Ca-P, in West lake was volatile and at the risk of P release. Additionally, the values of CEC and pH of lake sediments were 52.4 meq/100 g and 7.39, respectively.

### Adsorption capacities of various MMF samples

**Figure 1:**
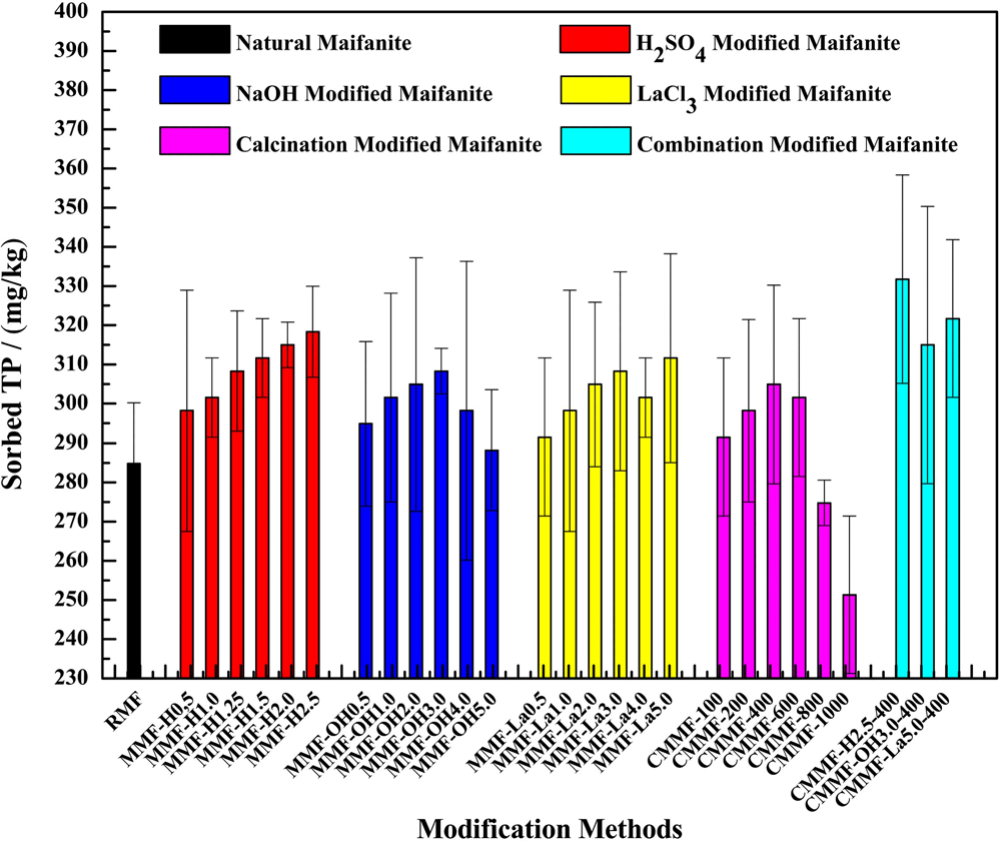
Effects of different RMF and MMF samples on adsorption of sediment TP.

Compared with the RMF, MMF samples exhibited higher adsorption capacities (Fig. 1). The modification could improve the adsorption capacity of RMF. MMF-H2.5, MMF-OH3.0, MMF-La5.0 and CMMF-400 were the optimal MMF samples among all acidification, alkalization, salinization and calcination MMF samples, respectively. The adsorption quantities of sediment TP by RMF, MMF-H2.5, MMF-OH3.0, MMF-La5.0 and CMMF-400 were 284.84 mg·kg^−1^, 318.35 mg·kg^−1^, 308.29 mg·kg^−1^, 311.644 mg·kg^−1^ and 304.94 mg·kg^−1^, respectively. The corresponding adsorption rates were 17.24%, 19.27%, 18.66%, 18.86%, and 18.46%, respectively. Based on these results, MMF-H2.5, MMF-OH3.0 and MMF-La5.0 samples were further calcined at 400 °C to obtain the combined MMF samples, named as CMMF-H2.5-400, CMMF-OH3.0–400 and CMMF-La5.0–400, respectively. The adsorption quantities of sediment TP by CMMF-H2.5-400, CMMF-OH3.0–400 and CMMF-La5.0–400 were 331.75 mg·kg^−1^, 314.99 mg·kg^−1^, and 321.7 mg·kg^−1^, respectively. The corresponding adsorption rates were 20.08%, 19.06% and 19.45, respectively. CMMF-H2.5-400 exhibited the highest adsorption capacity. Thus, CMMF-H2.5-400 was selected as the optimal adsorption material in the following experiments.

### Characterization of RMF and MMF

#### XRF

The XRF results (Table 1) showed that there were significant differences in the chemical compositions of RMF and various MMF samples, which were mainly composed of SiO_2_, Al_2_O_3_, Na_2_O, CaO and Fe_2_O_3_. The modification treatment resulted in an obvious increase in the content of SiO_2_ and a significant decrease in MgO and P_2_O_5_ of MMF samples. Typically, SiO_2_ and Al_2_O_3_ were the major compositions of these maifanite samples. Compared to the RMF, the content of Na_2_O in MMF-OH3.0 was increased. It indicated Na ion from 3.0 mol/L NaOH was intercalated in the layers of the RMF after the NaOH modification. This was also confirmed by XRD analyses.

**Table 1:** The main chemical compositions of (a) RMF, (b) MMF-H2.5, (c) MMF-OH3.0, (d) MMF-La5.0, (e) CMMF-400 and (f) CMMF-H2.5-400 (wt.%).

Sample	SiO_2_	Al_2_O_3_	Na_2_O	CaO	Fe_2_O_3_	MgO	K_2_O	TiO_2_	P_2_O_5_	Loss on ignition
a	61.38	16.26	5.16	5.08	3.18	1.82	1.74	0.36	0.22	4.38
b	61.98	15.35	5.01	4.79	3.07	1.48	1.88	0.35	0.18	4.48
c	61.78	15.92	5.79	4.73	3.41	1.72	1.81	0.37	0.20	3.83
d	63.51	15.74	5.08	4.25	3.10	1.26	1.91	0.31	0.20	3.58
e	61.64	16.31	5.31	5.90	3.08	1.79	1.77	0.30	0.19	3.40
f	62.44	15.91	5.31	4.41	2.88	1.54	1.80	0.39	0.17	3.75

#### FTIR

**Figure 2:**
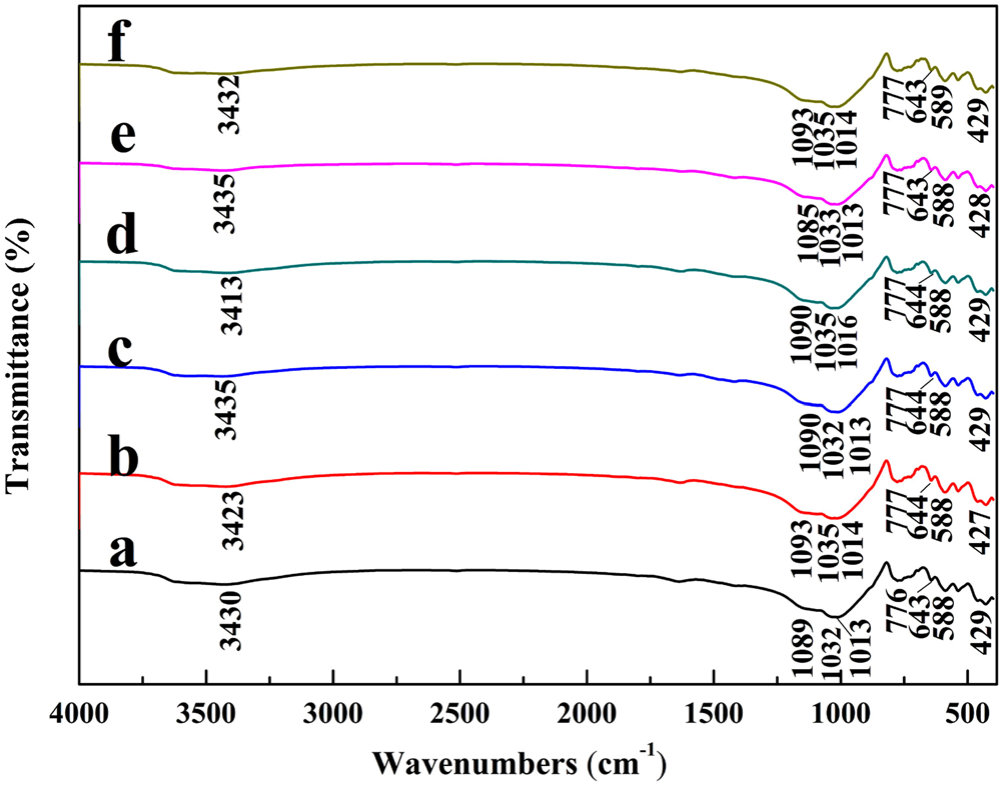
FTIR spectra of (**a**) RMF (**b**) MMF-H2.5 (**c**) MMF-OH3.0 (**d**) MMF-La5.0 (**e**) CMMF-400 and (**f**) CMMF-H2.5-400.

**Figure 3:**
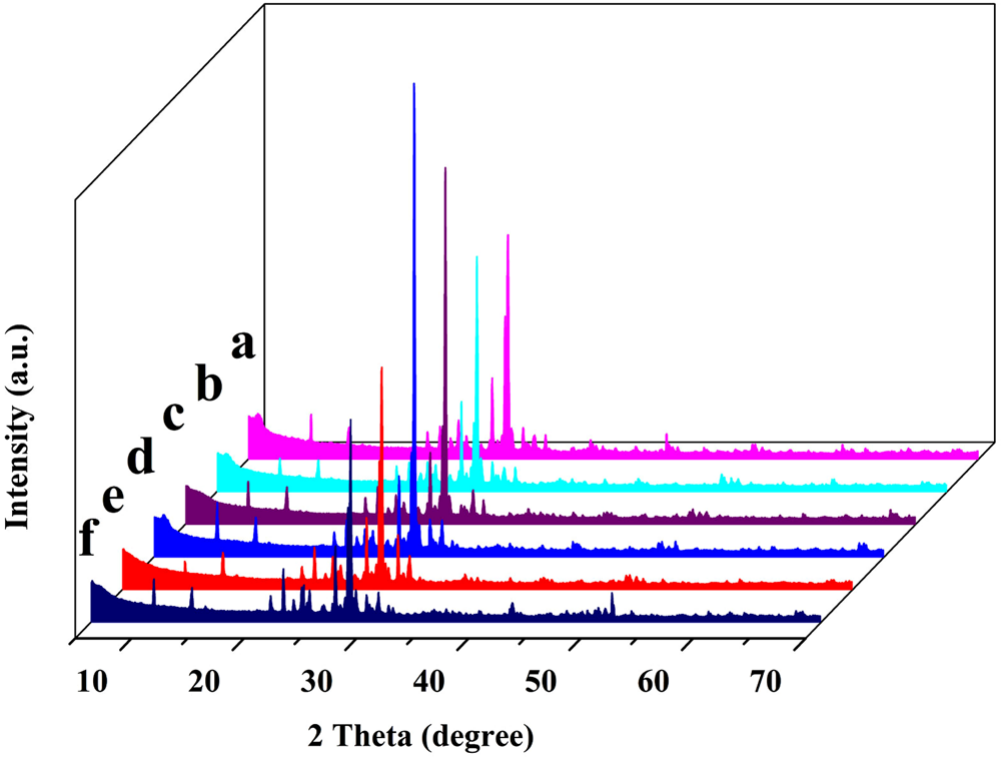
XRD patterns of (**a**) RMF (**b**) MMF-H2.5 (**c**) MMF-OH3.0 (**d**) MMF-La5.0 (**e**) CMMF-400 and (**f**) CMMF-H2.5-400.

FTIR is a supporting tool to distinguish characteristic functional groups^38,39^. In the spectrum of RMF and MMF samples (Fig. 2), a broad intense peak near 3430 cm^−1^ was due to the stretching vibrations of O-H and N-H groups^39^. The broad peak at 3430 cm^−1^ in RMF was shifted to 3423 cm^−1^, 3435 cm^−1^, 3413 cm^−1^, 3435 cm^−1^ and 3432 cm^−1^ in MMF-H2.5, MMF-OH3.0, MMF-La5.0, MMF-400, and CMMF-H2.5-400, respectively, suggesting that interfacial interaction between chemical reagent of the modification treatments and maifanite surface, and this was consistent with XRD results (Fig. 3). In the FTIR spectrum of the various maifanite samples, a prominent band at 1035 cm^−1^ could be assigned to a fundamental frequency of the PO_4_^3−^ stretching^40^. The peak at 777 cm^−1^ was assigned to Mg-Fe-OH^41^, and it remained unchanged after the modification treatments. The peak at 589 cm^−1^, probably assigned to the Fe-O bands^42^, was obviously unchanged, although with a low intensity, in the MMF samples.

#### XRD

XRD analyses of various maifanite samples were carried out to discuss the crystal structure and identity. XRD patterns (Fig. 3) revealed that the RMF and various MMF samples mainly contained Quartz (SiO_2_) and Margarite (CaAl_2_(Si_2_Al_2_)O_10_(OH)_2_). Additionally, it is obvious that the *d*_001_ value increased after the modification treatment (Table 2), and this result was agreement with the previous results^43^. Comparing the intensity of obvious diffraction peaks of RMF with MMF samples, there was no significant change. No obvious peaks of metal oxide were observed in the maifanite diffractograms, which indicated that the maifanite structures remained intact after the modification treatment and the metal oxide was well dispersed on the surface of maifanite^44^. XRD patterns (Fig. 3(a,e,f)) of maifanite before and after calcining at 400 °C indicated that their main mineral diffraction peak position remained unchanged. It indicated that 400 °C was the appropriate temperature on calcination. The result was in accordance with that of Yang *et al*. (2011), who reported that the main mineral diffraction peak position of maifanite did not change after calcining at 500 °C^45^. On the other hand, some peaks were disappeared and weakened a little after the modification treatment, which might be caused by the decrease of impurity after the modification treatment. This was consistent with BET and FTIR results.

**Table 2:** pH_PZC_, CEC, *d*_001_, surface and pore parameters of (a) RMF, (b) MMF-H2.5, (c) MMF-OH3.0, (d) MMF-La5.0, (e) CMMF-400 and (f) CMMF-H2.5-400.

Sample	pH_PZC_	CEC (meq/100 g)	*d*_001_ (nm)	S_BET_ (m^2^/g)	S_external_ (m^2^/g)	V_t_ (cm^3^/g)	V_mikro_ (cm^3^/g)	D_p_ (nm)
a	5.26	9.89	3.07	3.57	3.57	0.008	—	8.96
b	7.18	18.74	3.43	20.14	15.34	0.023	0.0018	4.57
c	7.59	20.12	3.28	18.37	12.67	0.025	0.0020	5.44
d	7.89	26.38	3.57	20.26	16.31	0.021	0.0019	4.15
e	6.31	16.74	3.21	17.13	13.42	0.027	0.0023	6.31
f	8.24	37.26	3.73	42.61	35.03	0.019	0.0016	1.78

#### SEM

**Figure 4:**
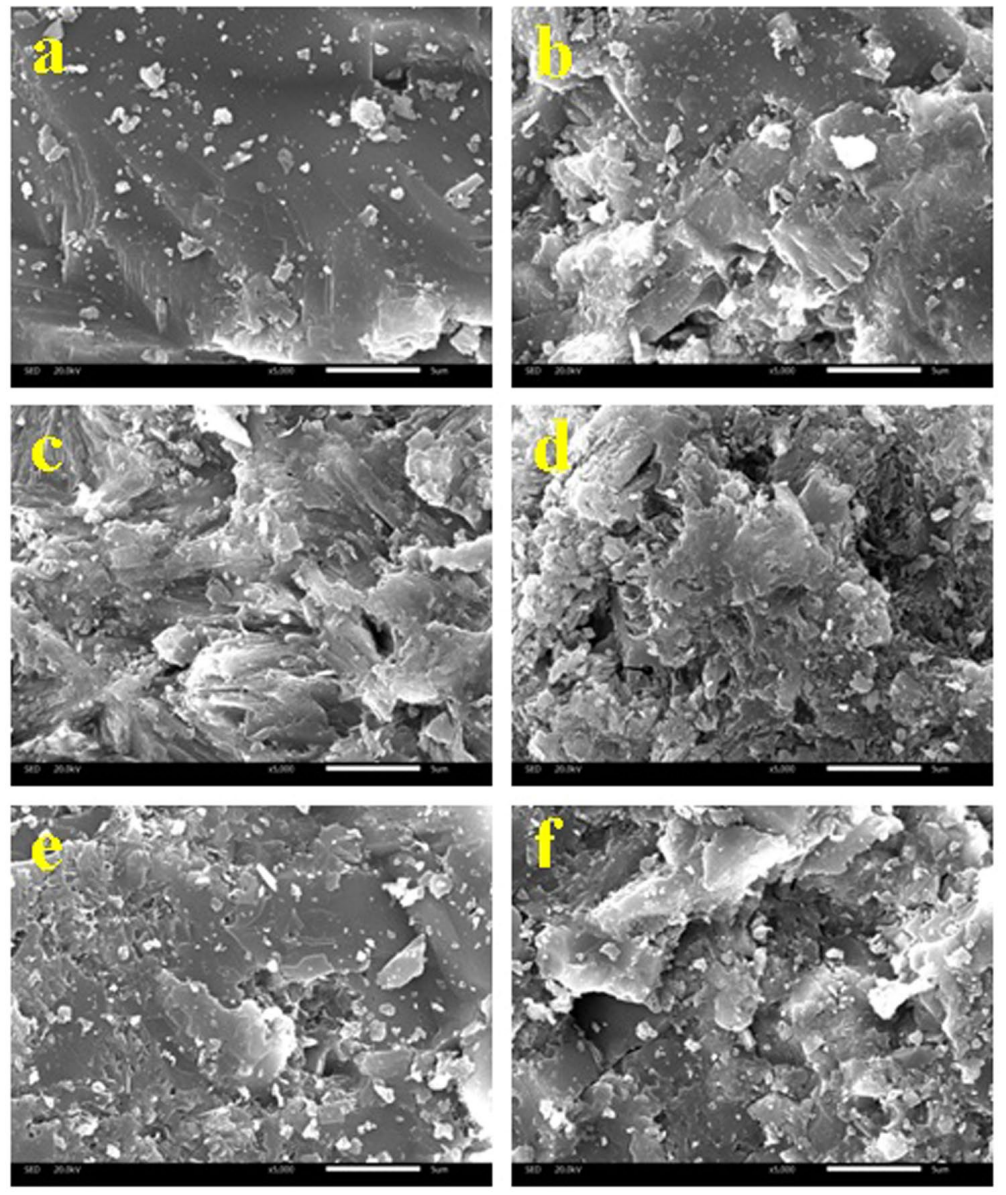
SEM micrographs of (**a**) RMF (**b**) MMF-H2.5 (**c**) MMF-OH3.0 (**d**) MMF-La5.0 (**e**) CMMF-400 and (**f**) CMMF-H2.5-400.

To compare the morphology of RMF with MMF samples, the SEM analysis were performed. It was seen that the morphology of MMF samples changed obviously due to the modification treatment (Fig. 4). The surface of the RMF was smooth, and few pores were observed. Comparing with the RMF, the surface of the MMF samples was rougher and more pores were obtained, which indicated that a porous structure with irregularly defined channel was formed. Additionally, there were more flakes appeared in MMF samples. The SEM micrographs of MMF-H2.5 (Fig. 4b), MMF-OH3.0 (Fig. 4c), MMF-La5.0 (Fig. 4d) and CMMF-H2.5-400 (Fig. 4f) showed that the activation of MMF samples resulted in smaller grain sizes caused by the dispersive effect of H^+^, Na^+^, La^3+^, respectively, on the MMF-H2.5, MMF-OH3.0, MMF-La5.0, and CMMF-H2.5-400 structures^46–48^. Calcination led to microporous, followed by the removal of surface water, bound water and water of hydration of CMMF-400 (Fig. 4e) and CMMF-H2.5-400 (Fig. 4f)^49,50^. These changes could improve the reactivity of maifanite and impart a higher capacity of adsorption.

#### Surface analysis

Generally, the BET equation has been applied to measure and compare the specific surface areas of a variety of porous materials^51^. The BET surface area was regarded as an important factor in determining the pore properties of the adsorbent materials^42^. The pH_PZC_, CEC, the maifanite basal plane diffractions *d*_001_, the specific surface (S_BET_), the total pore volume (V_t_), the volume of micropores (V_mikro_), the external surface (S_external_) and the average pore size (D_p_) before and after the modification were given in Table 2. The results suggested that the modification caused the disintegration of maifanite structural. Based on these, it led to a significant increase in the S_BET_ and an obvious decrease in the D_p_. It could provide more active sites for the adsorption reaction, and made the surface more available for the sediment P. These findings supported the SEM results. The pH_PZC_ of sorbents depended on various factors, including crystallinity nature, Si/Al ration, operation temperature and sorption capacity of the electrolytes, contents of impurity and degree of H^+^ and OH^−^ ions adsorption, etc. Thus, it might vary from adsorbent to adsorbent. Chutia *et al*.^52^ revealed that pH_PZC_ had a significant impact in As(V) adsorption by natural zeolites. That’s because the adsorption of multivalent cation occurred effectively at a pH below pH_PZC_. Compared to the RMF, the pH_PZC_ values of MMF-H2.5, MMF-OH3.0, MMF-La5.0, CMMF-400 and CMMF-H2.5-400 were increased in different degrees, respectively. On the other hand, The CEC of MMF samples was found to be higher than RMF, and the CEC of CMMF-H2.5-400 was the maximum. The above information revealed that modification treatments could improve the sediment P adsorption capacity of maifanite.

### P adsorption on maifanite by dynamic experiments

#### Effect of maifanite dosage

**Figure 5:**
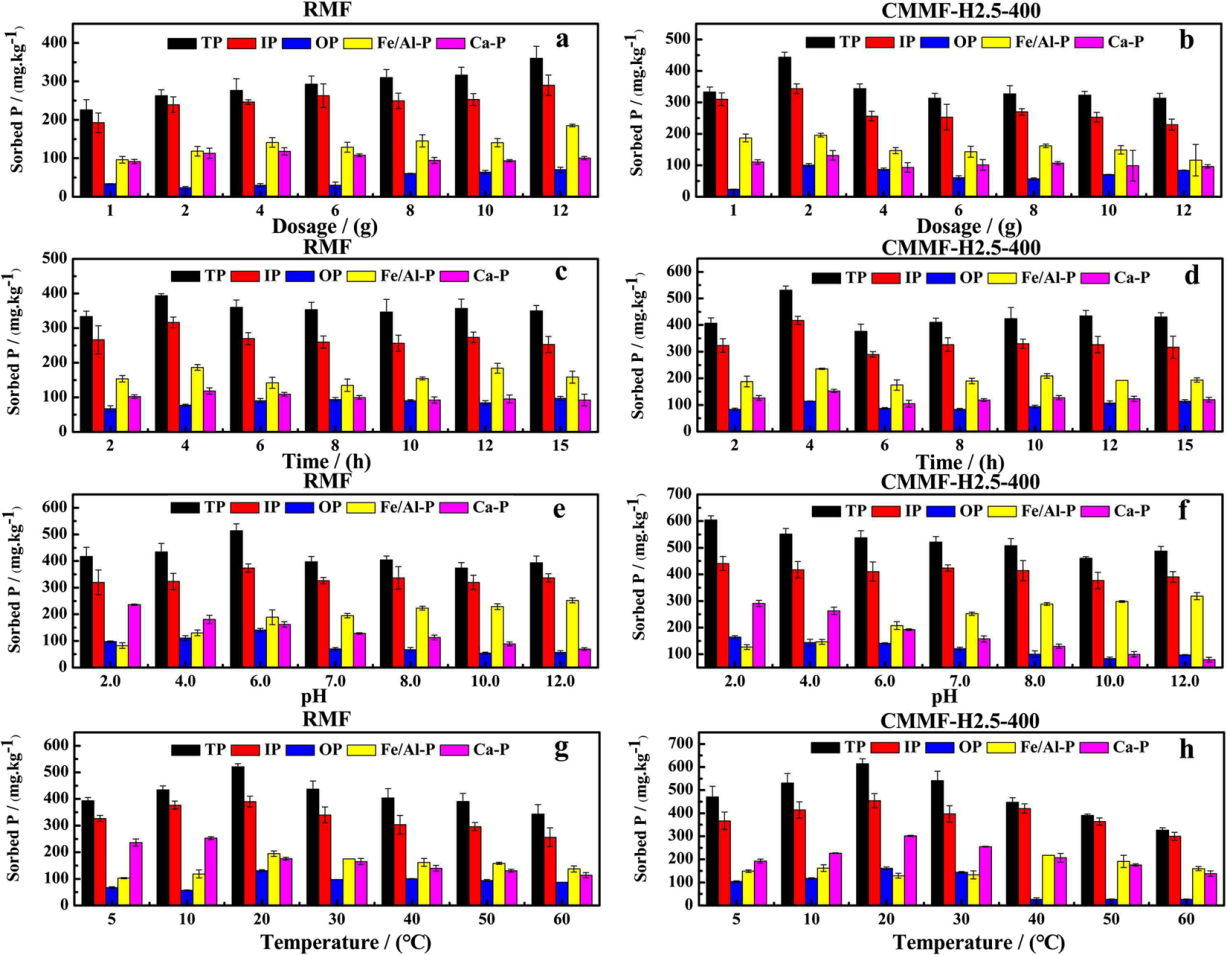
Effects of (**a**,**b**) dosage (**c**,**d**) time (**e**,**f**) pH and (**g**,**h**) temperature to the adsorption of sediment P in all fractions.

The amount of the sorbent dosage plays a vital role in sediment P adsorption. Experiments were carried out with various dosages of RMF and CMMF-H2.5-400, respectively at 20 ± 2 °C, pH 7.0 ± 0.2 and shaken at 200 rpm for 12 h to investigate the effects of maifanite dosage. Figure 5a,b indicated that the adsorption effects on sediment P by CMMF-H2.5-400 were better than RMF, with the tendency of increased at first then decreased and stabilized with an increase of dosage (Fig. 5b). Compared to the RMF, CMMF-H2.5-400 showed more considerable micropore adsorption and higher ion exchange capacity due to the more microporous microstructure and superior charge number^31^. Figure 5a indicated that the adsorption quantity of P from the sediment increased with an increase of RMF dosages. The quantity of adsorption sites becomes more availability with an increase of sorbent dosage^53,54^. From Fig. 5b, the adsorption quantity of P first increased and then decreased with the increasing of CMMF-H2.5-400 dosages. Typically, the adsorption of P didn’t have an immense change when CMMF-H2.5-400 was more than 4 g. This might be due to saturation between CMMF-H2.5-400 and P ^55^. The highest adsorption quantities for RMF and CMMF-H2.5-400 were found at 12 g and 2 g, respectively. The adsorption amounts of TP, IP, OP, Fe/Al-P and Ca-P by CMMF-H2.5-400 (2 g dosage) were 444.01 mg·kg^−1^, 343.44 mg·kg^−1^, 100.56 mg·kg^−1^, 195.87 mg·kg^−1^ and 131.53 mg·kg^−1^, respectively. The corresponding adsorption rates were 26.87%, 33.60%, 15.96%, 39.61% and 26.32%, respectively. The adsorption amounts of TP, IP, OP, Fe/Al-P and Ca-P by RMF (12 g dosage) were 360.23 mg·kg^−1^, 289.83 mg·kg^−1^, 70.41 mg·kg^−1^, 185.31 mg·kg^−1^ and 100.53 mg·kg^−1^, respectively. The corresponding adsorption rates were 21.80%, 28.35%, 11.18%, 37.48% and 20.12%, respectively. Based on the above results, optimal dosages were fixed as 2 g and 12 g, respectively for CMMF-H2.5-400 and RMF and pursued in further investigations.

#### Effect of stirring time

To investigate the effects of stirring time on P adsorption by maifanite, the experiments were carried out with 2 g CMMF-H2.5-400 or 12 g RMF at 20 ± 2 °C, pH 7.0 ± 0.2 and shaken at 200 rpm. Figure 5c,d presented the results. After the initial rapid reaction, the adsorption quantities of sediment P by CMMF-H2.5-400 or RMF decreased gradually. The P adsorption amounts by CMMF-H2.5-400 and RMF respectively increased from 2 h to 4 h and then decreased after 4 h. The adsorption amount of P from sediment reached the maximum at 4 h by CMMF-H2.5-400 and RMF, respectively. Thus, 4 h was chosen as the optimal stirring time to investigate the effects of pH and operation temperature on the P adsorption by CMMF-H2.5-400 and RMF, respectively. Especially, the adsorption quantities of IP and Fe/Al-P by CMMF-H2.5-400 and RMF increased markedly (P < 0.05), respectively.

#### Influence of pH

The operation pH is a key factor that controls the P adsorption from sediments^56^. To investigate the influence of extremely high or low pH value on adsorption of sediment P, the experiments were carried out at different pH conditions ranged from 2.0 to 12.0. Figure 5e,f confirmed that the operation pH influenced the adsorption capacity: the adsorption of TP by CMMF-H2.5-400 reached the maximum at the optimal pH (pH_opt_) 2.0 and decreased with the increasing pH value. The adsorption of TP by RMF increased with the increasing pH (2.0–6.0), then decreased when pH exceeded 6.0 (P < 0.05). The adsorption amounts of TP, IP, OP, Fe/Al-P and Ca-P by CMMF-H2.5-400 at pH_opt_ 2.0 were 604.86 mg·kg^−1^, 440.62 mg·kg^−1^, 164.23 mg·kg^−1^, 126.67 mg·kg^−1^ and 290.70 mg·kg^−1^, respectively. The corresponding adsorption rates were 36.61%, 43.10%, 26.07%, 25.62% and 58.16%, respectively. The adsorption amounts of TP, IP, OP, Fe/Al-P and Ca-P by RMF at pH_opt_ 6.0 were 514.38 mg·kg^−1^, 373.60 mg·kg^−1^, 140.78 mg·kg^−1^, 188.83 mg·kg^−1^ and 161.69 mg·kg^−1^, respectively. The corresponding adsorption rates were 31.13%, 36.55%, 22.35%, 38.19% and 32.35%, respectively. When the pH value increased from 2.0 to 12.0, the adsorption quantity of Ca-P by CMMF-H2.5-400 and RMF, respectively, decreased immensely (P < 0.05) while the adsorption amount of Fe/Al-P was the highest in alkaline conditions (P < 0.05). These results were agreement with the previous reports^31,57^. The effects of P adsorption in various pH were due to a series of mechanisms, including chemical interaction, ligand exchange, electrostatic attraction/repulsion and coagulation/precipitation^58^.

#### Effect of temperature

Operation temperature is a significant factor influencing the P adsorption in sediment, which could remarkably improve p release^59,60^. Shallow lakes are usually isothermal, and the sediment is susceptible to temperature variations^61^. Thus, the temperature may have a larger influence on adsorption P in sediment from shallow lakes. The results were depicted in Fig. 5g,h. As temperatures increased from 5 °C to 20 °C, the P adsorption efficiencies of CMMF-H2.5-400 and RMF, respectively, increased remarkably (P < 0.05). High temperatures facilitated the P adsorption confirming that the sediment P adsorption on the maifanite samples was an endothermic reaction. This result was agreement with the previous reports^61,62^. The adsorption amounts of TP, IP, OP, Fe/Al-P and Ca-P by CMMF-H2.5-400 at 20 °C were 614.91 mg·kg^−1^, 454.03 mg·kg^−1^, 160.88 mg·kg^−1^, 129.01 mg·kg^−1^ and 301.59 mg·kg^−1^, respectively. The corresponding adsorption rates were 37.22%, 44.41%, 25.54%, 26.09% and 60.34%, respectively. On the other hand, the adsorption amounts of TP, IP, OP, Fe/Al-P and Ca-P by RMF at 20 °C were 521.08 mg·kg^−1^, 390.36 mg·kg^−1^, 130.72 mg·kg^−1^, 194.69 mg·kg^−1^ and 175.93 mg·kg^−1^, respectively. The corresponding adsorption rates were 31.54%, 38.18%, 20.75%, 39.38% and 35.20%, respectively. Additionally, the adsorption quantities of P in all fractions decreased in various degrees above 20 °C. The results could be explained that the process of desorption as well as the Brownian movement were enhanced due to the high temperature^59,60^.

### Static adsorption experiments

**Figure 6:**
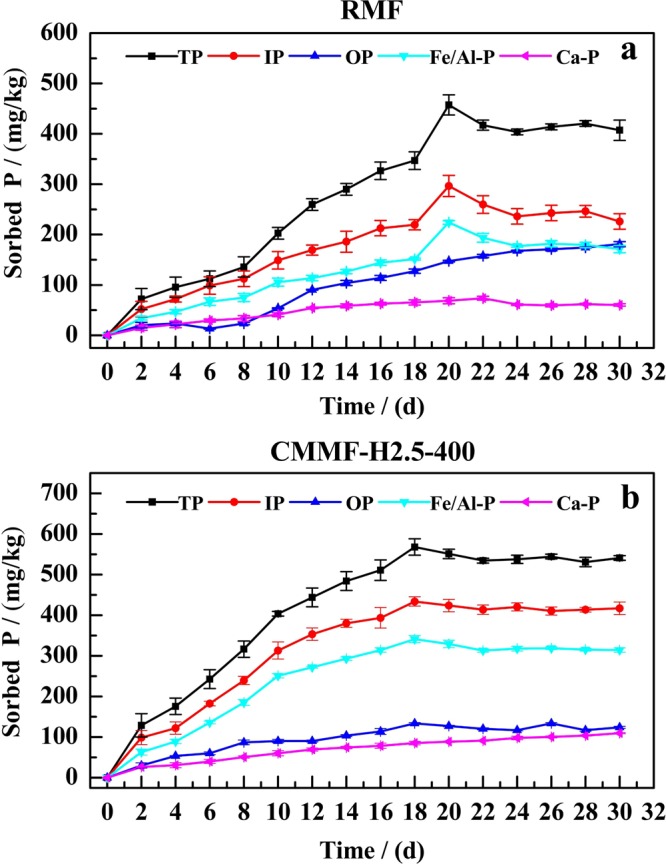
Effects of static time on adsorption performance of RMF (**a**) and CMMF-H2.5-400 (**b**).

To simulate the P adsorption under *in-situ* treatment, static experiments were carried out with time from 0 d to 30 d. Figure 6 depicted the adsorption of sediment P by RMF and CMMF-H2.5-400, respectively, under static conditions. The P adsorption on CMMF-H2.5-400 included quick, slow and dynamic balance adsorption steps (P < 0.05). Firstly, the quick adsorption step mainly occurred from 0 d to 10 d, and then followed by a slower second step (10 d–18 d). Furthermore, there was no obvious difference in adsorption quantities of P in all fractions from the sediment after 18 d (Fig. 6b). The adsorption quantity of sediment P by CMMF-H2.5-400 reached the peak value at 18 d. On the other side, the amounts of P adsorption on RMF increased rapidly within 20 d and then decreased slowly. Finally, the adsorption amounts of P barely achieved to a real equilibrium within the selected time (P < 0.05). The adsorption quantities of sediment P by RMF reached the maximum at 20 d (Fig. 6a). The adsorption amounts of TP, IP, OP, Fe/Al-P and Ca-P by CMMF-H2.5-400 at 18 d were 568 mg·kg^−1^, 433.92 mg·kg^−1^, 134.07 mg·kg^−1^, 341.30 mg·kg^−1^ and 85.45 mg·kg^−1^, respectively. The corresponding adsorption rates were 34.38%, 42.45%, 21.28%, 69.03% and 17.10%, respectively. On the other hand, the adsorption quantities of TP, IP, OP, Fe/Al-P and Ca-P by RMF at 20 d were 457.41 mg·kg^−1^, 296.53 mg·kg^−1^, 147.48 mg·kg^−1^, 224.01 mg·kg^−1^ and 68.70 mg·kg^−1^, respectively. The corresponding adsorption rates were 27.68%, 29.01%, 23.41%, 45.31% and 13.75%, respectively. Figure 6 also showed that the P adsorption capacity of CMMF-H2.5-400 was higher than RMF. Furthermore, it indicated that the modification treatment could improve the P adsorption capacity of maifanite, and this result was in agreement with our previous study, which reported that the P adsorption capacity of modified bentonite granules (MBGs) was higher than raw bentonite granules (RBGs)^31^.

### Characterization of maifanite after adsorption

**Figure 7:**
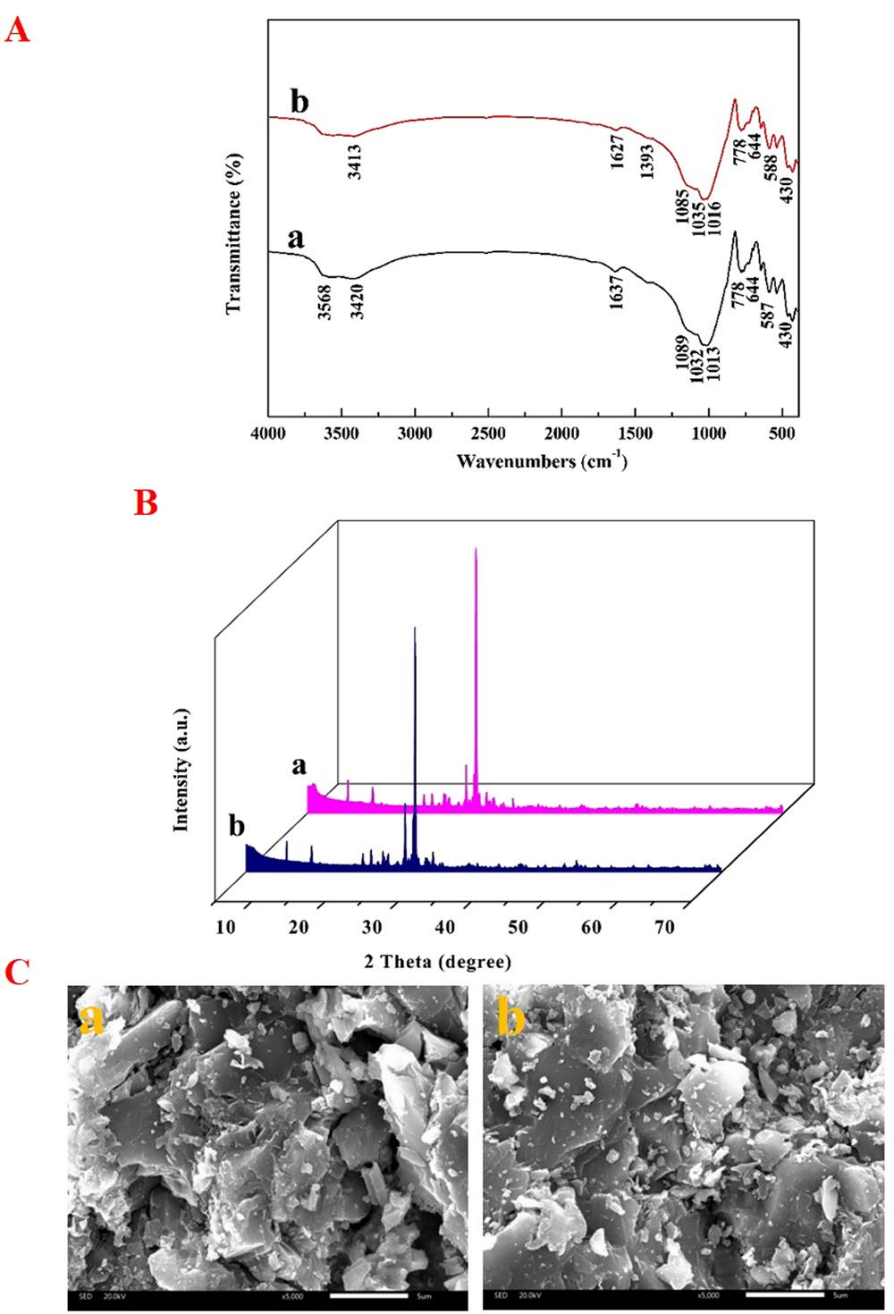
(**A**) FTIR spectra of (a) RMF and (b) CMMF-H2.5-400 after adsorption. (**B**) XRD patterns of (a) RMF and (b) CMMF-H2.5-400 after adsorption. (**C**) SEM micrographs of (a) RMF and (b) CMMF-H2.5-400 after adsorption.

Ion-exchange occurred and altered the elements content of CMMF-H2.5-400 and RMF, respectively, during the adsorption process (Table 3). The contents of SiO_2_, Fe_2_O_3_, MgO, K_2_O, TiO_2_ and P_2_O_5_ were increased respectively. However, the contents of Al_2_O_3_, CaO, Na_2_O and P_2_O_5_ were decreased respectively. Figure 7(A) depicted the FTIR spectra of CMMF-H2.5-400 and RMF after adsorption. Comparing the spectrum of CMMF-H2.5-400 after adsorption with that of CMMF-H2.5-400 before adsorption (Fig. 2b), a new adsorption peak emerged at 1393 cm^−1^, which referred to the O-H bending vibration with Fe (III), Al (III) species present on the surface of CMMF-H2.5-400 after adsorption^51^. It indicated that Fe (III), Al (III) species were intercalated into interlayers of CMMF-H2.5-400 by adsorption process. Additionally, the peaks of CMMF-H2.5-400 at 3432 cm^−1^, 1634 cm^−1^ and 1093 cm^−1^ were shifted to 3413 cm^−1^, 1627 cm^−1^ and 1085 cm^−1^, respectively. For the RMF, a new adsorption peak emerged at 3568 cm^−1^, which were attributed to broad OH-stretching in RMF after adsorption. On the other hand, the peaks of RMF at 3430 cm^−1^ and 776 cm^−1^ were shifted to 3420 cm^−1^ and 778 cm^−1^, respectively. Comparing the Fig. 7(A) with Fig. 2, it could be seen that the peaks observed on the maifanite samples were obviously unchanged after adsorption. XRD patterns of RMF and CMMF-H2.5-400 after adsorption were shown in Fig. 7(B). Comparing the intensity of obvious diffraction peaks of maifanite before and after adsorption, a reasonable shifting of peaks was observed from 20 to 70° 2 theta indicating that the adsorption of sediment P on maifanite granules changed the peaks of chemical composition intensities on the maifanite granules. According to the XRD spectra, it was thought that there was a ligand exchange between sediment P in all fractions and maifanite granules. The results of XRD in Figs 3 and 7(B) clearly reveal the presence of phosphate salts on the structure of adsorbent material after the adsorption process^63^. The *d*_001_ values of CMMF-H2.5-400 and RMF were 3.73 nm and 3.07 nm, respectively, before adsorption (Table 2), and then increased to 3.91 nm and 3.13 nm after adsorption, respectively (Table 4). SEM images were used to examine the surface morphology of RMF and CMMF-H2.5-400 before and after sediment P adsorption, respectively. Figure 7(C) exhibited the SEM images of RMF and CMMF-H2.5-400 after adsorption. Comparing the SEM images of maifanite samples before and after adsorption, some rough exterior and fresh cavities emerged after adsorption. The micrograph obtained after adsorption indicated that the flakes of the phosphate were observed on the adsorbent surface (Fig. 7(C))^64,65^. Furthermore, the pores of the particles of the adsorbent have been covered with adsorbate^63^. After the adsorption process, the SEM micrographs of CMMF-H2.5-400 and RMF revealed the formation of metal-hydroxyl-phosphate ligand (Yang *et al*., 2009). Table 4 confirmed that adsorption caused the disintegration of maifanite structural and led to a significant increase in the S_BET_. These findings supported the SEM results.

**Table 3:** The main chemical compositions of (a) RMF and (b) CMMF-H2.5-400 after adsorption (wt.%).

Sample	SiO_2_	Al_2_O_3_	Na_2_O	CaO	Fe_2_O_3_	MgO	K_2_O	TiO_2_	P_2_O_5_	Loss on ignition
a	61.87	15.84	5.07	4.75	3.34	1.95	1.85	0.39	0.19	4.28
b	63.43	15.47	4.86	4.17	3.05	1.68	1.83	0.43	0.12	3.88

**Table 4:** *d*_001_, surface and pore parameters of (a) RMF and (b) CMMF-H2.5-400 after adsorption.

Sample	*d*_001_ (nm)	S_BET_ (m^2^/g)	S_external_ (m^2^/g)	V_t_ (cm^3^/g)	V_mikro_ (cm^3^/g)	D_p_ (nm)
a	3.13	5.04	5.04	0.009	—	7.14
b	3.91	47.36	40.17	0.016	0.0013	1.36

### Adsorption mechanisms

Phosphate is adsorbed onto clay minerals via electrostatic, ligand exchange, and Lewis acid-base interaction^66–68^. Surface hydroxyl groups are protonated in the ligand exchange process at low pH. That’s because, compared to the hydroxyl groups, -OH_2_^+^ is easier to displace from the metal binding sites^43^. Therefore, it was likely that the adsorption of phosphate onto CMMF-H2.5-400 mainly using ligand exchange. This result was in agreement with our previous study, which reported that MBG adsorbed phosphorus mainly by anionic coordination exchange adsorption^31^. On the CMMF-H2.5-400 samples, phosphate replaced the hydroxyl groups, which were then released into the solution. The adsorption of phosphate could be speculated to take place as follows: the phosphate in the sediment was first transferred to the sites on the adsorbent; then, chemical complexation/ion exchange occurred at the active sites^69,70^.

Comparing the SEM images of the CMMF-H2.5-400 before and after adsorption process, we observed an aggregated morphology and some large flakes in CMMF-H2.5-400 samples after adsorption process. Additionally, the size of the intra-particle voids was decreased due to a stacking structure formed by some thin lamellas (Table 4). These results revealed that phosphate did adsorb onto the CMMF-H2.5-400 surface and it could be combined in the form of oxygen bridge^43,63^. Meanwhile, the hydroxyl and hydration base could be swop out^31^.

## Conclusions

In this study, MMF samples were prepared by various modification methods and applied to adsorb sediment P in all fractions for the first time. The results revealed that the modification treatment could improve the adsorption capacity of maifanite and CMMF-H2.5-400 was selected as the optimal *in-suit* adsorption material. The results of adsorption experiments showed that the dosage of maifanite, adsorption time, operation pH and operation temperature were the main factors influencing the adsorption performance on sediment P of CMMF-H2.5-400. Under the optimal conditions of dynamic experiments, the adsorption rates of TP, IP, OP, Fe/Al-P and Ca-P by CMMF-H2.5-400 were 37.22%, 44.41%, 25.54%, 26.09% and 60.34%, respectively. The adsorption mechanisms analyses revealed that the adsorption of phosphate onto CMMF-H2.5-400 mainly by ligand exchange. The above information indicated that CMMF-H2.5-400 exhibited a promising adsorption capacity on sediment P and could be further applied to reduce internal P loading in the eutrophic lakes. Furthermore, a combined technology of MMF and other ecological methods would be a significative orientation to treat sediment P.

## Methods

### Study site and sampling

West Lake (30°14′45′′N, 120°08′30′′E) is located on the western side of Hangzhou City, China. West Lake, which has been listed in the World Heritage Site in 2011, is a typical eutrophic lake with an area of 6.5 km^2^ and a mean depth of 2.27 m^30^. The sediment in West Lake is unstable, at the risk of releasing P to West lake^71^. Currently, developing and applying an effective *in-situ* technology for sediment P control was urgent.

The sampling site is located in a severe eutrophic region (30°23′16′′N, 120°13′18′′E) in Xiaonan Lake, one sub lake of West Lake. The surface lake sediments, at a depth of 0–10 cm, were collected by a Peterson grab sampler (model HNM1-2) on May 14th, 2017. The sediment samples were then stored in plastic bags. After transportation to the laboratory, the sediment samples were air-dried. The content of TP, TN, NH_4_^+^-N and NO_2_-N of overlying water above sediments in the lake was 0.06 ± 0.01 mg/L, 3.57 ± 0.03 mg/L, 0.15 ± 0.02 mg/L and 0.12 ± 0.01 mg/L, respectively. The pH values of the interstitial water and overlying water were 8.07 and 7.9, respectively.

### Preparation of MMF

RMF (3–5 mm in diameter, gray-white colour) granules were purchased from Kangyuan maifanite factory in mengyin country, Shandong Province, China. Firstly, the RMF granules were soaked in deionized water for 10 h and then washed with deionized water until the pH value reached 7.0. After that, the samples were dried at 105 °C for 24 h. Finally, the samples were cooled to room temperature for further studies, which were used as RMF. To improve the sediment P adsorption capacity of RMF, acidification, alkalization, salinization, calcination and combined modifications were used to modify the RMF granules, respectively. The preparation of various MMF samples was listed in Table 5.

**Table 5:** Preparation of different MMF samples.

Modification methods	Preparation of MMF samples
Acidification	Firstly, the RMF granules were mixed at 90 °C for 2 h with 0.5, 1.0, 1.25, 1.5, 2.0, and 2.5 mol/L H_2_SO_4_ solution, respectively, at liquid/solid of 3 mL/g in the thermostatic water bath. The samples were then washed by deionized water until the pH value reached 7.0. After that, the obtained granules were dried at 105 °C for 24 h. Finally, the acidification MMF samples were cooled to room temperature for further studies. The acidification MMF samples were named as MMF-H0.5, MMF-H1.0, MMF-H1.25, MMF-H2.0, and MMF-H2.5, respectively.
Alkalization	Firstly, the RMF granules were mixed at 90 °C for 2 h with 0.5, 1.0, 2.0, 3.0, 4.0, and 5.0 mol/L NaOH solution, respectively, at liquid/solid of 3 mL/g in the thermostatic water bath. The samples were then washed by deionized water until the pH value reached 7.0. After that, the obtained granules were dried at 105 °C for 24 h. Finally, the alkalization MMF samples were cooled to room temperature for further studies. The alkalization MMF samples were named as MMF-OH0.5, MMF-OH1.0, MMF-OH2.0, MMF-OH3.0, MMF-OH4.0, and MMF-OH5.0, respectively.
Salinization	Firstly, the RMF granules were mixed at 90 °C for 2 h with masses fraction of 0.5%, 1.0%, 2.0%, 3.0%, 4.0%, and 5.0% LaCl_3_ solution, respectively, at liquid/solid of 3 mL/g in the thermostatic water bath. The samples were then washed by deionized water until the pH value reached 7.0. After that, the obtained granules were dried at 105 °C for 24 h. Finally, the salinization MMF samples were cooled to room temperature for further studies. The salinization MMF samples were named as MMF-La0.5, MMF-La1.0, MMF-La2.0, MMF-La3.0, MMF-La4.0, and MMF-La5.0, respectively.
Calcination	The RMF granules were calcined at 100, 200, 300, 600, 800, and 1000 °C, respectively, for 2 h. After that, the calcination MMF samples were cooled to room temperature for further studies. The calcination MMF were named as CMMF-100, CMMF-200, CMMF-300, CMMF-600, CMMF-800, and CMMF-1000, respectively.
Combined modifications	The optimal acidification modification, alkalization modification, salinization modification, and calcination modification methods were calcined at the optimal temperature, respectively. After that, the combined MMF samples were cooled to room temperature for further studies.

### Batch experiment

To explore the optimal modification method of RMF, 5 g sediment samples and 8 g various MMF samples were added to Erlenmeyer flasks, containing 250 mL 0.02 mol/L KCl solution with pH 7.0 ± 0.2, then shaken at 200 rpm for 12 h in a thermostatic shaker (Shanghai Boxun Medical Equipment Plant, China) at 20 ± 2 °C. After the adsorption process, the mixture was filtered through a steel screen (60 mesh) to remove maifanite granules. The sediment was then dried in an oven at 55 °C for 24 h. Each treatment consisted of three replicates.

#### Dynamic adsorption experiments

Dynamic adsorption experiments were carried out in thermostatic bottle shakers. Typically, 250 mL KCl solution (0.02 mol/L) was poured into Erlenmeyer flasks, and then 5 g sediment samples and different dosages (1 g, 2 g, 4 g, 6 g, 8 g, 10 g and 12 g) of maifanite samples (RMF and MMF, respectively) were added into the Erlenmeyer flasks. After that, they were shaken at 200 rpm for certain hours (2 h, 4 h, 6 h, 8 h, 10 h, 12 h and 15 h) at different pH (2.0–12.0). The dynamic adsorption experiments were carried out at temperatures of 5 °C, 10 °C, 20 °C, 30 °C, 40 °C, 50 °C, and 60 °C. Two drops of 0.1% chloroform were added in each Erlenmeyer flask to inhibit bacterial activity.

#### Static adsorption experiments

Static adsorption experiments were carried out in Erlenmeyer flasks with pH 7.0 ± 0.2 in the dark condition for 0–30 d at 20 ± 2 °C. A certain quantity of maifanite (12 g RMF and 2 g CMMF-H2.5-400, respectively) and 5 g sediment samples were added to Erlenmeyer flasks. Two drops of 0.1% chloroform were added in each Erlenmeyer flask to inhibit bacterial activity.

### Analytic methods

The Standards and Measurements and Testing (SMT) protocol^72^ were used for determining P fractions. The P fractions can be characterized as TP (total P), IP (inorganic P), OP (organic P), Fe/Al-P (P bound to Al, Fe, and manganese (Mn) oxides extracted by NaOH) and Ca-P (P bound to calcium (Ca) extracted by HCl). The presence of metals in sediment can mediate the transport of P. Each P fraction concentration was measured directly using the ammonium molybdate spectrophotometric method with an UV-visible spectrophotometer at 700 nm (DR4000/U, HACH company, USA). The cation exchange capacity (CEC) of the sediment samples was analyzed using NH_4_Cl-NH_4_OAc method. Sediment pH was measured in 1:10 (w/v) solid/water suspensions by a PHS-3C digital pH meter (Shanghai LeiCi instrument plant, China).

The chemical compositions of RMF and MMF samples were determined by X-ray fluorescence (XRF, RU-200B/D/MAX-RB RU-200B, China). Fourier transform infrared (FTIR) spectra of the RMF and MMF samples were measured by a FTIR spectrometer (Nicolet6700, USA) in the wavenumber range of 400–4000 cm^−1^. The mineralogical and chemical compositions of the lake RMF and MMF samples were determined by X-ray diffractometer (XRD, RU-200B/D/MAX-RB RU-200B, Japan) operating with CuKα radiation (30 kV, 15 mA) over the range (2 Theta) of 5–70°. The microstructure of RMF and MMF samples was characterized by scanning electron microscope (SEM, JSM-5610LV, Japan). The CEC of maifanite samples, which was defined by the ability of maifanite granules to adsorb the cations, was analyzed using the ammonium acetate method. The specific surface areas were calculated by nitrogen adsorption using the Brunauer-Emmett-Teller (BET) equation on an analyzer (ASAP 2020 M, America). The t-plot method was applied to gain the volume of micropores and the surface of mesopores together with the external surface. The total pore volume was derived from the nitrogen volume adsorbed at the relative pressure p/p_0_ → 1 ^73^. The batch equilibrium techniques were applied to estimate the points of zero charge (pH_pzc_) of maifanite samples^52^.

All the chemicals and reagents used were analytical grade. All glassware and sample bottles were presoaked before use in diluted HCl solution for at least 12 h followed by washing with deionized water and drying in oven. Deionized water was used for preparing solutions.

### Data analysis

The amount of P adsorbed on the maifanite granules and the adsorbed efficiency (*A*) were calculated using the following equations:

1 $$q={q}_{0}-{q}_{e}$$

2 $${\rm{Adsorbed}}\,{\rm{efficiency}}( \% )=({q}_{0}-{q}_{e})/{q}_{0}\times 100$$

where *q* is the adsorption quantity of P per unit weight of maifanite samples (mg·kg^−1^), and *q*_0_ and *q*_e_ (mg·kg^−1^) are the initial and final P quantity, respectively.

All treatments were conducted in triplicate. OriginPro 8.0 (OriginLab Corporation, Northampton, MA, USA) was used to plot various figures. All statistical analyses were estimated using SPSS 18.0 (SPSS software, IBM, USA). Analyses of the variance (ANOVA, one factor) were applied to test the significant differences between the dependent variables (the adsorption quantity of P) and independent variables (the corresponding adsorption parameter). The difference was considered statistically significant when the significance level was smaller than 0.05.
